# Two-photon excited photoconversion of cyanine-based dyes

**DOI:** 10.1038/srep23866

**Published:** 2016-03-31

**Authors:** Sheldon J. J. Kwok, Myunghwan Choi, Brijesh Bhayana, Xueli Zhang, Chongzhao Ran, Seok-Hyun Yun

**Affiliations:** 1 Harvard Medical School and Wellman Center for Photomedicine, Massachusetts General Hospital, 50 Blossom Street, Boston, Massachusetts, 02114, USA; 2 Harvard-MIT Health Sciences and Technology, Massachusetts Institute of Technology, 77 Massachusetts Avenue, Cambridge, Massachusetts, 02139, USA; 3 Department of Biomedical Engineering, Sungkyunkwan University and Center for Neuroscience and Imaging Research, Institute for Basic Science, 2066 Seobu-ro, Jangan-Gu, Suwon-Si, Gyeong Gi-Do, South Korea; 4 Molecular Imaging Laboratory, MGH/MIT/HMS Athinoula A. Martinos Center for Biomedical Imaging, Building 149, 13^th^ Street, Charlestown, Massachusetts, 02129, USA; 5 Department of Pharmacy, Zhongda Hospital, Southeast University, Nanjing 210009, China

## Abstract

The advent of phototransformable fluorescent proteins has led to significant advances in optical imaging, including the unambiguous tracking of cells over large spatiotemporal scales. However, these proteins typically require activating light in the UV-blue spectrum, which limits their *in vivo* applicability due to poor light penetration and associated phototoxicity on cells and tissue. We report that cyanine-based, organic dyes can be efficiently photoconverted by nonlinear excitation at the near infrared (NIR) window. Photoconversion likely involves singlet-oxygen mediated photochemical cleavage, yielding blue-shifted fluorescent products. Using SYTO62, a biocompatible and cell-permeable dye, we demonstrate photoconversion in a variety of cell lines, including depth-resolved labeling of cells in 3D culture. Two-photon photoconversion of cyanine-based dyes offer several advantages over existing photoconvertible proteins, including use of minimally toxic NIR light, labeling without need for genetic intervention, rapid kinetics, remote subsurface targeting, and long persistence of photoconverted signal. These findings are expected to be useful for applications involving rapid labeling of cells deep in tissue.

Since the discovery of photoactivable green fluorescent protein (paGFP) in 2002^1^, considerable attention has been placed on finding and engineering various phototransformable reporters, particularly fluorescent proteins (FPs) for selective cell labeling and localization-based super-resolution microscopies^2^,^3^,^4^,^5^,^6^,^7^,^8^,^9^,^10^,^11^. Phototransformation of FPs is induced typically by one-photon absorption under ultraviolet or deep-blue radiation (>2.75 eV). Phototransformation can be irreversible or reversible. Photoactivatable or photoconvertible FPs have chromophore structures that undergo light-induced irreversible modification that result in either off-on activation (photoactivation) or shift in emission wavelength (photoconversion). One such mechanism in many photoconvertible proteins is peptide bond cleavage and extension of the π conjugated system causing a red-shift in fluorescence emission spectra^8^,^10^,^12^. Photoswitchable FPs are reversibly switched between a fluorescent-on and dark-off state through conformational changes such as cis-trans isomerization^13^,^14^,^15^. For cell tracking over long time periods, photoconvertible FPs are generally used since photoswitchable FPs relax spontaneously to their stable state (typically fluorescent) within minutes to hours^10^. Photoconvertible probes also enable both non-converted and converted cells to be distinguished and tracked simultaneously. However, photoconversion speed is rather slow^2^,^3^,^16^,^17^, typically 0.5 to 10 s per cell, and limited by poor photostability at higher powers as well as risk of phototoxicity to the target cells and surrounding tissues^2^,^3^,^18^.

Phototoxicity can be reduced by two-photon excitation at the NIR window, which also confines excitation to the focal plane, minimizing off-target phototoxicity. For example, one-photon activation of paGFP at 408 nm could not be accomplished without compromising cellular division, while two-photon activation at 820 nm maintained cellular viability up to a maximum peak irradiance^18^ of 1.5 × 10^12^ W/cm^2^. Despite the benefits of non-linear excitation, two-photon excited photoconversion of FPs has not been a viable option for cell tracking applications due to their low two-photon conversion efficiencies^17^,^19^,^20^,^21^,^22^,^23^. In addition, since FPs are genetically encoded, continued production of the unconverted protein and intrinsic protein turnover dilutes photoconversion contrast, limiting the time for which cells can be tracked.

Organic fluorophores are attractive alternatives to FPs as they are generally brighter and have higher photostability^9^,^24^,^25^. While they lack the target specificity of fluorescent proteins, fluorophores offer versatility for labelling cells without genetic intervention. Fluorophores are currently more suitable than FPs for use in primate models or clinical applications, for which genetic engineering is challenging. On the other hand, they may be used in conjunction with genetically encoded FPs designed to label specific proteins or subcellular compartments. Fluorophores can be synthetically combined with a photo-labile protecting group to form photoactivatable fluorophores (or caged dyes), which can be irreversibly activated from a non-fluorescent to fluorescent state by one or two-photon photolysis^26^,^27^,^28^,^29^,^30^. Polymethine cyanine dyes represent one family of bright and biocompatible fluorophores that can be reversibly photoswitched^31^,^32^,^33^. Photoswitching of cyanine dyes occurs by a light-catalyzed chemical reaction (typically UV) with reducing thiols and the polymethine chain of the cyanine dye, disrupting the π conjugated system^34^. Accordingly, this method requires the addition of a primary thiol, specific buffer conditions and an oxygen-free environment, which is generally incompatible with *in vivo* imaging^35^,^36^.

Here, we report our observation of photoconversion of cyanine-based fluorophores with femtosecond laser irradiation. This multiphoton process converts the dyes to a stable and fluorescent photoproduct in living systems without the need for additional reagents. This process is unique in three major aspects. First, it utilizes a novel mechanism that induces an irreversible blue-shift of fluorescence by shortening of the dye’s π conjugated system. Second, we observe two-photon conversion kinetics that are significantly faster than previously reported photoconvertible proteins. Finally, the use of nonlinear excitation for both photoconversion and imaging offers compelling advantages of low phototoxicity of NIR excitation light confined to the focal volume, which also enables precise three-dimensional control of photoconversion. Our findings are expected to be particularly useful for applications involving rapid labeling and tracking of cells in deep tissues.

## Results

### Two-photon photoconversion of cyanine-based dyes

**Figure 1:**
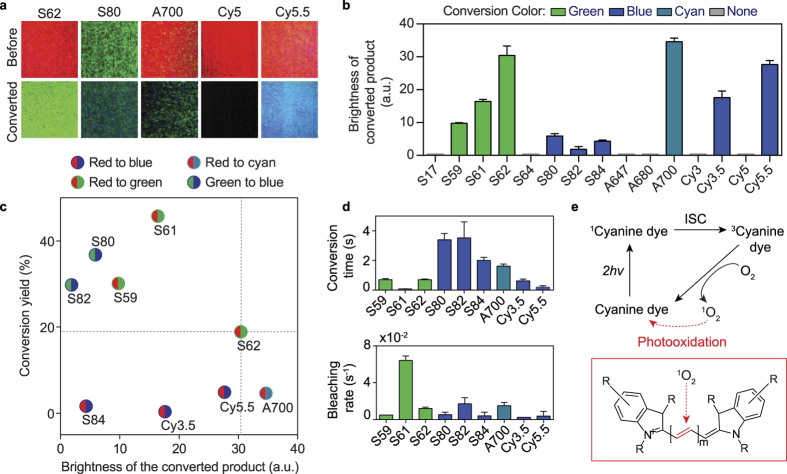
Multiphoton excited photoconversion of cyanine-based dyes. (**a**) Multiphoton excited fluorescence images of common red and green cyanine-dyes on glass slides before and after femtosecond irradiation at 810 nm. Images are normalized such that the initial brightness is similar. (**b–d**) Quantitative analysis of photoconversion properties. (**b**) Brightness of the converted product (ΔF) is defined as the maximum increase from baseline (in arb. units) before photobleaching in the blue, green or cyan channels. (**c**) Conversion yield plotted against brightness of the converted product for photoconverting dyes. Conversion yield is defined as the brightness of the converted product over the loss of the initial red or green fluorescence, when dyes are fully converted. (ΔF/ΔR_i_) (**d**) Conversion time (τ) and bleaching rate (k_1_) determined for each dye by fitting to *f*(t) = *f*_*max*_(1−*k*_1_t−*e*^−t/τ^), where f is either blue, green or cyan fluorescence depending on the dye. (**e**) Schematic of singlet-oxygen mediated photooxidation of cyanine dyes. ^1^Dye and ^3^Dye represent excited singlet and triplet states respectively. ISC: intersystem crossing.

Using a custom-built real-time two-photon microscope with a mode-locked Ti:sapphire laser (150 fs pulses at 80 MHz) (Supplementary Fig. S1), we tested fifteen commercially available cyanine-based dyes with one-photon absorption peaks ranging from 531 nm to 702 nm (Supplementary Table 1). Photoconversion and imaging were conducted simultaneously at a single 810 nm excitation wavelength, and all measurements were done with dyes alone in aqueous solution. Nine out of fifteen dyes had measurable blue-shifted photoconversion, including red to green, red to blue, green to blue and red to cyan (Fig. 1a,b). Photoconversion was irreversible in all cases, while prolonged illumination resulted in bleaching of the converted dye. We quantified the brightness of the converted product, conversion yield, speed of conversion, as well as the bleaching kinetics (Fig. 1b–d). The choice of dye depends on the application; for instance, high photoconversion yield is required for maximal ratiometric contrast, high brightness is required for imaging deep in tissue, and fast conversion speed is needed for photoconversion of rapidly moving cells. For most dyes, a region spanning (150 by 150) μm^2^ was photoconverted with less than 2 seconds total illumination (512 × 512 pixels, 7.6 μs pixel dwell time, 106 μs exposure time per spot size) with a scanning beam at 80 mW power or 6.7 MW/cm^2^ irradiance. This corresponds to a fluence level of 710 J/cm^2^, which is well below a DNA reported damage threshold^37^ of 5 × 10^4^ J/cm^2^.

**Figure 2:**
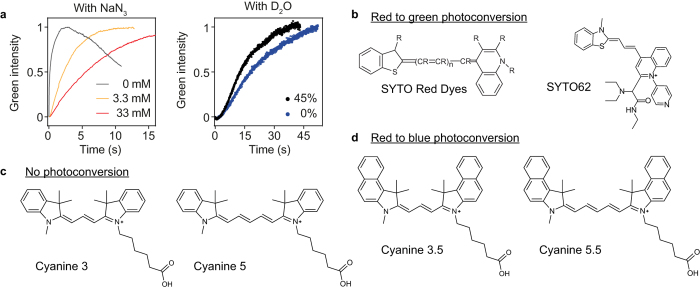
Chemical structures of photoconvertible cyanine dyes. (**a**) Photoconversion of SYTO62 in the presence of NaN_3_ (left) or D_2_O (right). The curves are normalized for visualization purposes; however, we observed quenching of the green fluorescence by ~63% with NaN_3_ (3.3 or 33.3 mM) and enhancement of the green fluorescence by ~31% with D_2_O (45%). (**b**) Left: The backbone structure of SYTO red dyes (US Patent 20090068672), for which red-to-green photoconversion was observed. Right: Proposed structure of SYTO 62 based on LC-MS (MW = 550 g/mol), ^1^H, ^13^C, gCOSY and DEPT NMR. Data on chemical analyses are available upon request. (**c**) Chemical structures of Cyanine 3 and Cyanine 5 (provided by Lumiprobe), for which photoconversion was not observed. (**d**) Chemical structures of Cyanine 3.5 and Cyanine 5.5 (provided by Lumiprobe), for which red-to-blue photoconversion was observed.

The primary photodegradative mechanism of cyanine dyes is dependent on singlet oxygen generated by energy transfer from excited triplet fluorophores to molecular oxygen^24^,^31^ (Fig. 1e). Singlet oxygen subsequently attacks the double bounds of the polymethine chain, breaking the π conjugations and fragmenting the dye to carbonyl products^38^. HPLC analysis of SYTO62 prior to and following photoconversion confirmed fragmentation of the dye accompanied by a shift in elution time from 23.3 to 15.1 min, respectively (Supplementary Fig. S2). We found that both photobleaching and photoconversion of SYTO62 is inhibited in the presence of sodium azide, a singlet oxygen scavenger. On the other hand, the photoconversion rate was enhanced by addition of D_2_O, which increases the lifetime of singlet oxygen (Fig. 2a). These results suggest that photoconversion involves the same, singlet oxygen-mediated photooxidative process. This irreversible process may be unique to cyanine dyes given the sensitivity of the polymethine chain to singlet oxygen attack and subsequent chemical cleavage^31^.

The efficiency of photoconversion likely depends on a number of factors, including absorption cross-section, intersystem crossing rate, oxidation potential, steric considerations and photostability of the photoproduct^38^. Photoconversion can be affected by the presence of specific functional groups, such as the R groups at the end of the polymethine chain, which influences both absorption cross-sections and photostability^38^. For instance, photoconvertible SYTO red dyes (e.g. SYTO61 and SYTO62) share a common quinoline group, which has favorable two-photon properties^39^ (Fig. 2b). However, not all cyanine dyes that underwent photobleaching by femtosecond excitation formed stable, fluorescent photoproducts. For example, while blue-shifted photoconversion was observed for Cy3.5 and Cy5.5, only bleaching was observed for Cy3 and Cy5. The only difference in chemical structure between Cy3 and Cy3.5 and between Cy5 and Cy5.5 is the addition of a fused benzene ring at each end of the heteroaromatic groups connected by the polymethine chain. (Fig. 2c,d). These observations suggest that specific R groups can play a critical role in enabling two-photon excited photoconversion.

### Spectroscopic analysis of SYTO62

**Figure 3:**
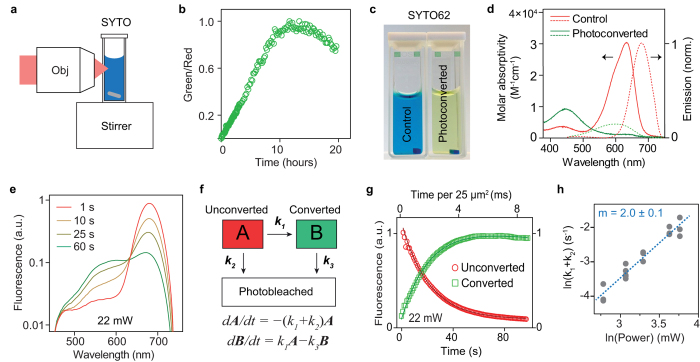
Optical characterization of SYTO62 photoconversion. (**a,b**) Photoconversion of a large volume for use for absorption spectroscopy. The sample was photoconverted with 15 hours laser irradiation under stirring. (**c**) Photographs of a SYTO62 solution-containing sample before and after large-volume photoconversion. (**d**) Solid line: absorption spectra of the unconverted and converted samples. Dotted line: emission spectra of the unconverted and converted computed using non-negative matrix factorization. (**e**) Representative spectral kinetics over time during photoconversion. (**f**) Kinetic model describing photoconversion and photobleaching assuming first-order kinetics, where A and B represent unconverted and converted dye respectively. (**g**) Representative fitting of the components A and B. (**h**) Log-log plot of the rate constant vs. excitation power fits to a line with slope of 2.0 ± 0.1.

Among the dyes we tested, we found that SYTO62, a cell permeable nucleic acid stain, had among the highest conversion yield and brightness of the converted product (Fig. 1c). SYTO dyes are also known to have enhanced quantum yields of nearly 40-fold when bound to nucleic acids, making them ideal for cell labeling applications^40^. The absorption peak of SYTO62 blue-shifts after photoconversion, which was also apparent by the change in color (Fig. 3a–d). This observation supports that photoconversion is a photodegradative process involving shortening of the dye’s π conjugated system^41^. Time-resolved two-photon excited fluorescence spectroscopy of SYTO62 during photoconversion revealed two distinct emission peaks in the red and green wavelengths (Supplementary Fig. S3). The bimodal spectrum was readily decomposed using non-negative matrix factorization to two components: ‘A’ (unconverted, centered at 679 nm) and ‘B’ (converted, centered at 600 nm) (dotted line, Fig. 3d). Photoconversion provided ratiometric contrast (converted/unconverted) of over 400 (Supplementary Fig. S4). The kinetics of photoconversion was modeled by first-order reactions (Fig. 3e,f). Global fitting of the kinetic data yielded excellent fits, with R^2^ > 0.96 for all spectra (Fig. 3g). The rate constant for photoconversion scaled quadratically (n = 2.0 ± 0.1) with excitation power, suggesting photoconversion indeed occurs through a two-photon absorption mediated process (Fig. 3h).

### Photoconversion of SYTO62 stained cells

**Figure 4:**
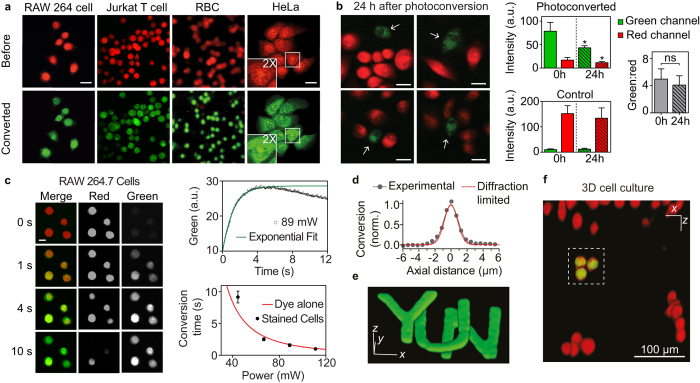
*In vitro* photoconversion and depth-resolved photoconversion. (**a**) Fluorescence images of SYTO62 stained cells, including HeLa, RAW 264.7, Jurkat T, and murine red blood cells (RBC), before and immediately after photoconversion. Scalebar, 20 μm. (**b**) Left: Quantification of green and red fluorescence channels for photoconverted and unconverted control HeLa cells at 0 and 24 hours following photoconversion and re-plating. Right: Ratiometric contrast (green:red) for photoconverted cells is maintained after re-plating and 24 hours incubation. (**c**) Left: Real-time photoconversion of stained RAW 264.7 cells. Scalebar, 10 μm. Right: Kinetic analysis of *in vitro* photoconversion. Conversion time was obtained by single exponential fitting of the green intensity until the onset of photobleaching. Plotted red line is the predicted dependence of conversion time on power obtained from dye alone in Fig. 3h. (**d**) Measurement of photoconversion axial resolution (FWHM, 2.3 ± 0.1 μm) compared to theoretical diffraction-limited resolution (FWHM, = 1.9 μm). (**e**) Three letters ‘Y’, ‘U’, and ‘N’ written at different planes separated by ~8.5 μm in dye-mixed, transparent cured optical epoxy. Scalebars in x, y, z, 20 μm. (**f**) Selective photoconversion of EL4 cells at depth in three-dimensional hydrogel. The dotted white box shows selected volume that was photoconverted.

One advantage of using chemical probes over genetically encoded proteins is versatility for loading into any cell type without transfection^5^. We performed two-photon photoconversion and imaging of various cell lines *in vitro*, including HeLa, RAW 264.7, Jurkat T and murine red blood cells (RBCs) (Fig. 4a). Photoconverted cells could also be readily visualized with one-photon confocal microscopy (Supplementary Fig. S5). There was no noticeable acute leakage of the dye during photoconversion. However, we observed changes in subcellular distribution (Fig. 4a,b). Mostly notably, while the original form of SYTO62 preferably stained the nucleus, 24 hours after photoconversion there was enhanced staining of the cytoplasm relative to the nucleus presumably due to a change in the dye’s binding specificity. We also found that photoconversion affected intracellular retention time. Following photoconversion, HeLa cells were trypsinized, re-suspended and imaged again at 24 hours (Fig. 4b). The fluorescence of photoconverted dye decreased 45% (p < 0.0001) at 24 hours, compared to a smaller 12% decrease of the red fluorescence in unconverted cells (p = 0.0043) which suggests increased leakage or decreased chemical stability of the photoconverted dye.

Nevertheless, the ratiometric contrast (green/red) of the photoconverted cells was not changed significantly at 24 hours, providing a mean to track the labeled cell over at least 24 hours. This is in stark contrast to photoconvertible FPs, such as Kaede, for which intrinsic protein turnover and continued synthesis of unconverted protein leads to halved ratiometric contrast at 6 hours and near reversion to the original fluorescence color by 24 hours^4^. Neither cellular staining by SYTO62 (10 nM-10 μM) nor photoconversion affected cell viability at 24 hours (Fig. 4b, Supplementary Fig. S6). The photoconversion rate of stained RAW 264.7 cells at different laser powers matched our spectroscopic analysis of SYTO62 alone, suggesting photoconversion kinetics are unchanged by binding to nucleic acids and the cellular environment (Fig. 4c and Supplementary Movie S1).

A major advantage of photoconversion through two-photon excitation is selective remote targeting in three-dimensions^19^,^42^. This intrinsic sectioning capability enabled selective labeling of subsurface EL4 cells cultured in a three-dimensional hydrogel^43^ (Fig. 4d). The measured axial resolution of photoconversion with 810 nm excitation was 2.28 ± 0.10 μm, close to theoretical diffraction-limited resolution (1.90 μm)^44^ (Fig. 4e). The small discrepancy (~20%) is attributed to systematic optical aberrations and saturated absorption. Finally, we showed optical 3D writing at near diffraction-limited resolution in a non-diffusive bulk polymer (Fig. 4f and Supplementary Movie S2). Future applications of two-photon excited photoconvertible dyes could include three-dimensional, high-density optical data storage in biological material.

## Discussion

We have presented two-photon excited photoconversion of cyanine-based fluorescent dyes. Two-photon excitation enables depth-resolved photoconversion with non-toxic NIR light. The intensity-squared dependence of two-photon excited photoconversion permits faster conversion times at lower overall energy deposition or fluence. This is in contrast to one-photon photoconversion, for which conversion time is inversely proportional to irradiance; thus, energy deposition is fixed. We found that some cyanine dyes could also be photoconverted with one-photon irradiation (Supplementary Fig. S7), but only at much higher power and fluence levels (1.2 × 10^4^ J/cm^2^). This suggests photoconversion yield may depend on the excitation mode (one or two-photon), which can have different selection rules and bleaching mechanisms^45^,^46^.

Two-photon photoconversion of SYTO62 is significantly faster than previously reported photoconvertible FPs. A recent study showed that two-photon photoconversion is negligible for many popular photoconvertible FPs^17^ using 800 nm, 4.3 MW/cm^2^ and 191 μs per spot size (or 818 J/cm^2^), which is comparable to the laser parameters used in our study (710 J/cm^2^ for Fig. 1). For EosFP, one of the most efficient photoconvertible proteins, two-photon photoconversion^47^ required at least 4 times longer exposure time than photoconvertible cyanine-based dyes presented in our study using the same laser power. In practice, two-photon phototransformation of FP requires at least several minutes of laser scanning per cell^23^, compared to tens of cells in less than a minute with SYTO62 (Fig. 4). The poor phototransformation efficiencies of FPs can be attributed to their low two-photon absorption cross-sections (2PA). A major challenge is the high sensitivity of the chromophore’s 2PA cross-section to local electric-field variations within the complex protein environment, which renders two-photon properties less predictable than one-photon properties, especially compared to free dyes in solution^48^. Aside from improved photoconversion efficiencies, cyanine-based dyes also offer a low-cost and versatile alternative for labeling cells, particularly in applications involving longitudinal tracking over days. However, for applications involving specific molecular labeling, additional chemical analyses are needed to determine the structure and properties of the photoconverted products. This information would facilitate chemical modification of these dyes such as addition of target moieties, and provide more desirable characteristics including higher quantum yields, improved photostability and faster kinetics.

Recently, continuous wave illumination at two wavelengths (blue and NIR) was used to efficiently photoconvert the FP Dendra2 through a novel, primed conversion mechanism^17^. While this method could be a viable alternative for applications not requiring the full axial resolution afforded by two-photon excitation, custom optical components are required and the need for visible blue light limits tissue penetration. Photoconvertible fluorophores are generally preferred over photoactivable or caged fluorophores, since both non-converted and converted populations can be analyzed simultaneously with ratiometric contrast^49^. Several photoconvertible dyes have been reported. In one study, Cy5 and Cy3 are conjugated with a UV-cleavable linker, creating a photoconvertible Cy5-Cy3 probe through resonance energy transfer^50^. DiR, a lipophilic membrane dye has been shown to photoconvert upon one-photon excitation with red light, which is attributed to an irreversible conformational change^5^. To our knowledge, the class of cyanine-based dyes described here are the only photoconvertible fluorophores suitable for multiphoton microscopy.

In our current method, photoconversion and imaging are conducted simultaneously at the same excitation wavelength from a single laser by changing optical power. This offers the practical benefit of real-time readout and control of photoconversion efficacy without the need for different conversion and imaging wavelengths, which is almost always necessary for one-photon convertible FPs and dyes^16^. However, this could be a disadvantage if imaging causes non-negligible photoconversion. In our study, imaging is typically conducted at low powers (≤10 mW) with minimal photoconversion (Fig. 4a,b). For example, at 3 mW average power, imaging can be performed for integrated imaging times of 5 min with <20% photoconversion. This imaging duration is sufficient in most applications for dynamic visualization of cell trafficking or intermittent time-lapse imaging over a day. Spectral separation of imaging and conversion modes may also be achieved by optimizing the excitation wavelength.

Our dye-based photoconversion approach allows persistent labeling of numerous cell types and longitudinal tracking of converted cells for over 24 hours. The rapid kinetics could enable photoconversion of circulating cells *in vivo* at single-cell resolutions, suitable for tracking circulating tumor cell migration^16^ or lymphocyte trafficking^19^. Given the interest in using two-photon microscopy for studying dynamic cellular processes^51^, we expect our results to be broadly useful for applications involving selective and rapid labeling of cells in three-dimensional tissue.

## Materials and Methods

### Two-photon microscopy

For imaging and photoconversion, we used a home-built, video-rate, two-photon microscope. The system equipped a mode-locked Ti-Sapphire laser (MaiTai DeepSee eHP, Newport) as a light source which provides ~150 fs width pulses at 80 MHz repetition rate. The scanning unit is composed of a polygon mirror for fast axis (*x*) and a galvanometer for slow axis (*y*), allowing frame rate of 30 Hz. One of the following water-immersion objectives (Olympus) was used: 40×, 0.8 NA or 20×, 0.9 NA. This system is designed to have a fixed field-of-view (FOV) of 150 × 150 μm^2^ with a 40× objective lens. The emitted light was detected by three photomultiplier (PMT) tubes through dichroic and bandpass filters for the blue channel (400–485 nm), green channel (500–550 nm) and red channel (560–705 nm). Cyan channel refers to the sum of the blue and green channels. The PMT gains were set such that detection of the blue-shifted photoproduct is ~10 dB higher than the unconverted product.

### Photoconversion of fluorescent dyes

For photoconversion of dyes alone, 3 μL of dye was mixed with 1 μL of 3 μm polystyrene beads (10^−4^% v/v) and dispensed on a glass slide, covered with a #1.5 coverslip and placed under the objective lens. The final concentration of SYTO dyes (Life Technologies) used (following dilution) was 3.75 mM. Alexa dyes (Life Technologies) were used at 7.5 g/L. Cy 5 (Lumiprobe) was used at 0.1 g/L. All other cyanine dyes (Lumiprobe) were used at 10 g/L. To quantify green enhancement from photoconversion, the sample was exposed to the femtosecond laser delivering 810 nm light at a power of 80 mW for up to 30 s. The bleaching of the initial red fluorescence (ΔR_i_) and signal enhancement in the green or blue channels by photoconversion (ΔF) were quantified to calculate conversion brightness (ΔF) and conversion yield (ΔF/ ΔR_i_).

### High-pressure liquid chromatography

HPLC analysis was performed on a C18 reverse phase column (250 mm *10 mm) using a diode array detector. The eluents were A = 0.1% TFA in water and B = 0.1% TFA in acetonitrile; the gradient method was from a relative concentration of 98.0% of A and 2.0% of B to 100% of B over a time of 40 min with a flow rate of 2.0 mL min.

### Spectroscopic analysis of photoconversion

To analyze two-photon fluorescence during photoconversion, we coupled emitted photons to a multi-mode optical fiber (0.39 NA, 1500 μm core; Thorlabs) connected to a spectrophotometer (Dongwoo). Excitation light is filtered out by a 750 nm dichroic mirror, and a 50:50 beamsplitter is used to enable both spectra and images to be taken simultaneously, which allows the focal plane to be easily visualized. For the photoconverted product of Cy3.5 and AlexaFluor700, an additional dichroic mirror at 560 nm was used to filter out the fluorescence of the unconverted dye.

For kinetic analysis of SYTO 62, 10 μL of SYTO 62 in DMSO was mixed with 10 μL of 1% agarose gel in distilled water to reduce diffusion of the sample during photoconversion. This sample was then mounted on a glass slide for spectroscopic analysis. Large volume (>2 ml) photoconversion of SYTO 62 was accomplished by overnight femtosecond irradiation of the sample in a quartz cuvette at 810 nm and 500 mW under vigorous stirring. Absorption spectra of the sample were taken prior and following photoconversion (Evolution 300, Thermo Scientific). Photoconversion of SYTO 62 was also performed in the presence of sodium azide or D_2_O (Sigma-Aldrich). For these experiments, 10 μL of 5 mM SYTO 62 in DMSO was mixed with appropriate amounts of sodium azide in water (final concentrations 5 mM and 50 mM) or D_2_O (total 45%).

### Spectral decomposition

We measured the kinetics of SYTO 62 photoconversion at excitation powers ranging from 16 mW to 43 mW using a spectrophotometer (Dongwoo) coupled to an emCCD (Andor). Analysis was done using MATLAB (Mathworks). Each spectra was smoothed (smoothed with a span of 30 nm) and decomposed into two components (*A* and *B*) using non-negative matrix factorization. These two components were fitted to a first-order kinetic model. The analytic solutions are presented below. For component *A* (concentration of unconverted dye):


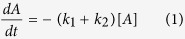



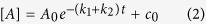


where *k*_1_ is photoconversion rate of *A*, *k*_2_ is photobleaching rate of *A*, and *c*_0_ is an offset. For component *B* (concentration of photoconverted dye):


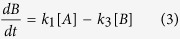



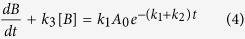



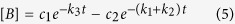


where is *k*_3_ is photobleaching rate of *B*, and *c*_1_ and *c*_2_ are constants. Assuming *k*_3_t ≪ 1,


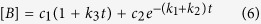


Global fitting of components *A* and *B* to the solutions (Eqs 2 and 6) was performed using Origin (Originlab), yielding the rate constant (*k*_1+_*k*_2_) for each experiment taken at each power. At least three experiments were done at each power.

### Cell culture and viability assay

HeLa cells and RAW 264.7 cells (ATCC) were maintained in Dulbecco’s modified Eagle’s medium (DMEM) supplemented with 10% fetal bovine serum and 1% antibiotics at 37 °C in 5% CO_2_. For cell viability assay, HeLa cells were stained with SYTO 62 at concentrations ranging from 1 nM to 10 μM. After 1 day, cells were stained with LIVE/DEAD viability assay kit (Life Technologies) and quantified using an inverted fluorescence microscope (IX71, Olympus).

### *In vitro* photoconversion

A typical protocol for photoconversion of cells *in vitro* involved exposing the cells to a femtosecond pulsed laser delivering light at 810 nm at 50 mW for up to 20 s. During photoconversion, the sample was moved up and down with a translational stage to ensure photoconversion of the entire volume.

### Photoconversion lifetime measurement

HeLa cells subcultured on a 60 mm dish were grown until confluent and stained with SYTO 62 (5 μM). The stained cells were imaged at 10 mW with exposure time of 3 s and then photoconverted at 50 mW for 10 s. By moving the sample with the translational stage, a rectangular area of ≈15 × 0.8 mm^2^ was photoconverted. Following photoconversion, unconverted cells in the surrounding area were gently removed with a cell scraper and the remaining cells (rectangular area of ≈40 × 3 mm^2^) were trypsinized. The trypsinized cells were then centrifuged, re-plated, and placed back into the incubator. After 24 hours, the cells were imaged again at 10 mW to measure the loss in fluorescence signal in photoconverted and unconverted cells.

### Axial resolution measurement

UV curable optical adhesive (NOA 81, Thorlabs) was mixed with SYTO 62 (0.5 mM), 10 μl was dispensed on the slide glass, and covered with a #1.5 coverslip. The sample was then cured with exposure to a UV lamp (365 nm, 5 mW/cm^2^; Spectroline) for 1 min. We targeted ~10 μm depth below the coverslip and photoconverted a single plane (50 mW, 1 min) with a water immersion objective lens (40×, NA 0.8). After photoconversion, the power was lowered to 10 mW and z-stack images were acquired from the surface to the 17 μm depth with 0.85 μm axial spacing (2 s integration for each image). The full-width-half-maximum (FWHM) of the photoconverted signal (green) along z axis was measured and deconvoluted with imaging resolution.

### 3D optical writing

We prepared a negative mask for the three letters (Y, U, N) using a laser printer and a transparent film. The mask was placed on a conjugate plane to the imaging plane between the scan lens and the tube lens. Using the sample containing 0.5 mM SYTO 62 in a cured optical adhesive, the photoconversion for each mask was performed with axial spacing of 8.5 μm between the letters (50 mW, 1 min for each letter). Photoconverted area in the sample was then imaged at 10 mW by z-stacking with 0.85 μm axial spacing. A 3D rendered image on was generated by ZEN (Carl Zeiss).

### Hydrogel fabrication

Hydrgel precursor solution was prepared by mixing PEGDA (10% w/v; Laysan Bio), 0.05% w/v photoinitiator Irgacure 2959 (Ciba), and EL4 cells (10^5^ cells/ml). The precursor solution was transferred to a 60 mm culture dish, and exposed to an ultraviolet lamp (365 nm, 5 mW/cm^2^; Spectroline) for 15 min to induce gelation. The crosslinked hydrogel was placed in culture medium containing SYTO 62 to stain the encapsulated cells for 1 hour. The hydrogel was washed twice by replacing the culture medium.

### Data analysis

ImageJ (NIH) and Matlab was used for image processing and data quantification. Data was presented as mean ± standard deviation unless otherwise stated and *p*-values lower than 0.05 were considered statistically significant.

## Additional Information

**How to cite this article**: Kwok, S. J. J. *et al.* Two-photon excited photoconversion of cyanine-based dyes. *Sci. Rep.* **6**, 23866; doi: 10.1038/srep23866 (2016).

## Supplementary Material

Supplementary Information

Supplementary Movie S1

Supplementary Movie S2
