# Supplementary material for: Two-photon excited photoconversion of cyanine-based dyes
Source: Sci Rep. 2016 Mar 31;6:23866. doi: 10.1038/srep23866 (PMC4814926; doi:10.1038/srep23866)
Supplement: Supplementary Information [file srep23866-s1.pdf]

Supplementary Information

Two-photon excited photoconversion of cyanine-based dyes

Sheldon J.J. Kwok[1,2*], Myunghwan Choi[1,3*],

Brijesh Bhayana[1], Xueli Zhang[4,5], Chongzhao Ran[4], and Seok-Hyun Yun[1,2#]

[1]Harvard Medical School and Wellman Center for Photomedicine, Massachusetts General Hospital, 50 Blossom Street, Boston, Massachusetts, USA, 02114.

[2]Harvard-MIT Health Sciences and Technology, Massachusetts Institute of Technology, 77 Massachusetts Avenue, Cambridge, Massachusetts, USA, 02139.

[3]Global Biomedical Engineering, Sungkyunkwan University and Center for Neuroscience and Imaging Research, Institute for Basic Science, 2066 Seobu-ro, Jangan-Gu, Suwon-Si, Gyeong Gi-Do, South Korea

[4]Molecular Imaging Laboratory, MGH/MIT/HMS Athinoula A. Martinos Center for Biomedical Imaging, Building 149, 13th Street, Charlestown, Massachusetts, USA 02129.

[5]Department of Pharmacy, Zhongda Hospital, Southeast University, Nanjing 210009, China.

*These authors contributed equally to this work.

[#] Corresponding Author:

S. H. Andy Yun, Ph.D.

Associate Professor, Harvard University

65 Landsdowne St. UP-525, Cambridge, MA 02139, USA

Tel: 1-617-768-8704, Email: syun@hms.harvard.edu

Supplementary Table S1	p. 2
Supplementary Figures S1 – S7	pp.3-6
Supplementary Movies S1 – S2	p. 7

Supplementary Table: Screening of commercially available cyanine-based dyes

Fluorophore	Absorption peak (nm)	Emission peak (nm)	Observed photoconversion
SYTO80	531	545	G→B
SYTO82	541	560	G→B
Cyanine3	555	570	None
SYTO84	567	582	R/G→B
Cyanine3.5	591	604	R→B
SYTO64	599	619	None
SYTO17	621	634	None
SYTO59	622	645	R→G
SYTO61	628	645	R→G
Cyanine5	646	662	None
AlexaFluor647	650	665	None
SYTO62	652	676	R→G
AlexaFluor680	684	707	R→G
Cyanine5.5	673	707	R→B
AlexaFluor700	702	723	R→G/B

Table S1 | Screening of fifteen commercially available cyanine-based dyes. One-photon absorption and emission maxima are obtained from the vendors (Life Technologies for SYTO and AlexaFluor; Lumiprobe for cyanine dyes). R, red. G, green. B, blue.

Supplementary Figures

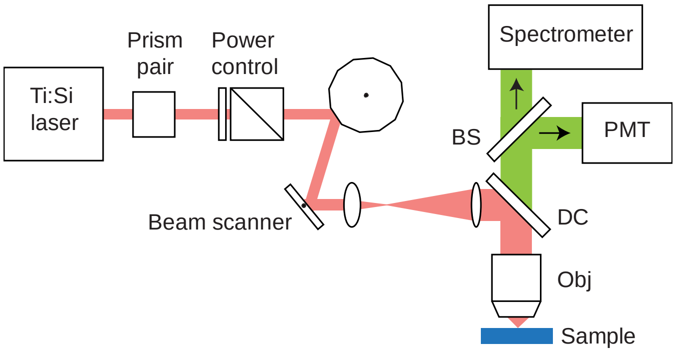

Figure S1 | Optical Setup.

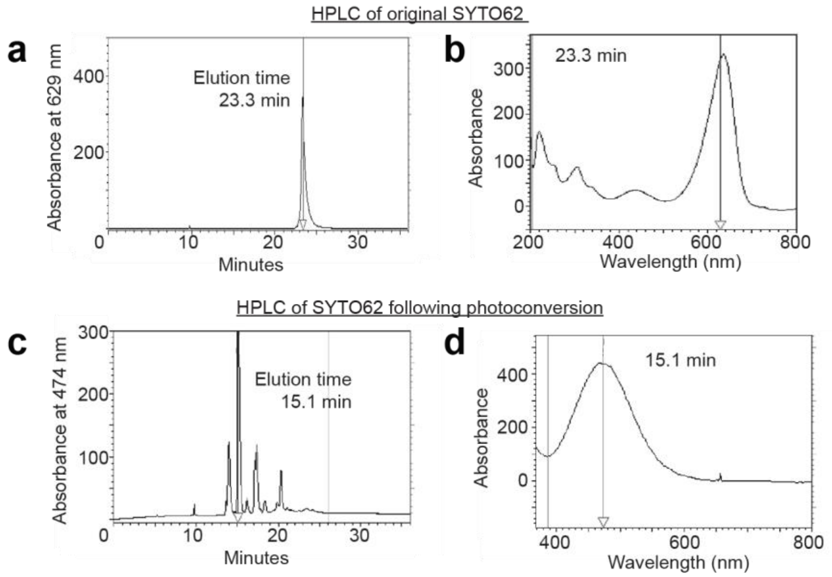

Figure S2 | High performance liquid chromatography analysis of SYTO62 photoconversion. **a-b**, Original SYTO62: **a**, Absorbance at 629 nm as a function of elution time. **b**, Absorption spectra of the compound eluted at 23.3 min. **c-d**, Photoconverted SYTO62: **c**, Absorbance at 474 nm as a function of elution time. **d**, Absorption spectra of the compound eluted at 15.1 min.

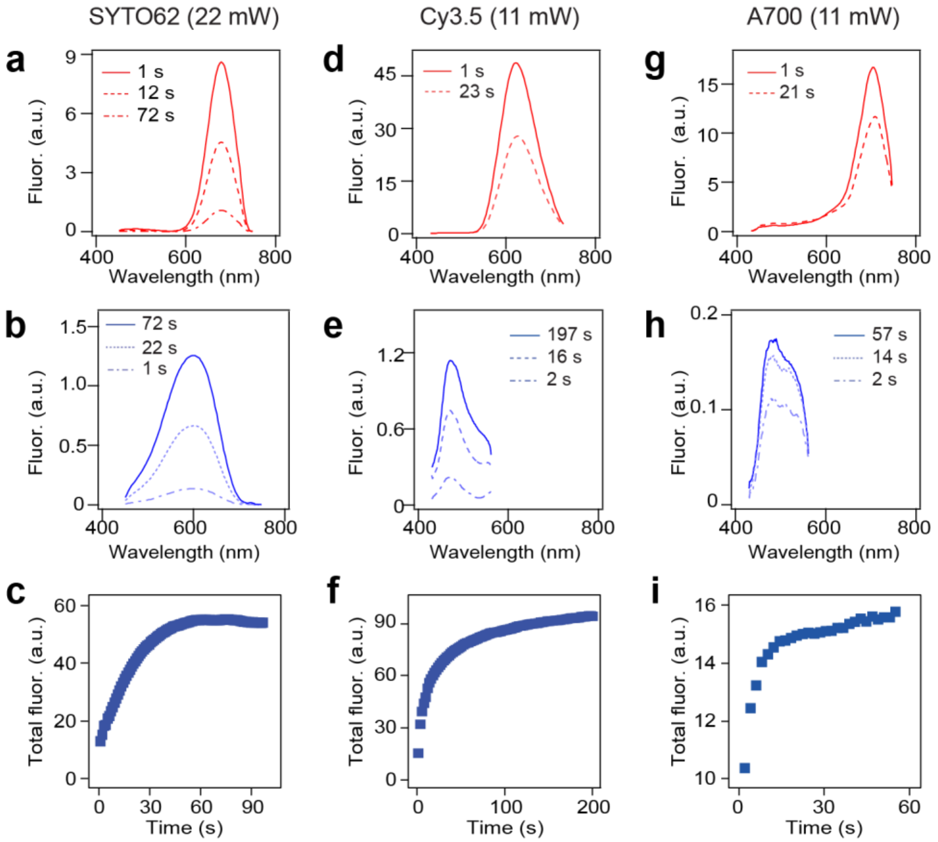

Figure S3 | Spectral characterization of photoconversion for several red fluorescent dyes. a-c, SYTO62: **a**, Decrease in the red fluorescence of the unconverted component (computed, component A) over time. **b**, Increase in the green fluorescence of the converted component (component B) over time. **c**, Total fluorescence over time, obtained by integrating data shown in (b). **d-f**, Cyanine 3.5. **d**, Decrease in red fluorescence over time (raw data). **e**, Increase in fluorescence over time. **f**, Total fluorescence over time, obtained by integrating data shown in (e). **g-i**, AlexaFluor 700: **g**, Decrease in red fluorescence over time (raw data). **h**, Increase in fluorescence over time. **i**, Total fluorescence over time, obtained by integrating data shown in (h).

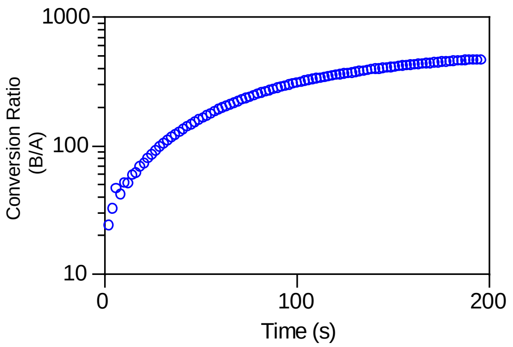

Figure S4 | Ratiometric contrast with photoconversion. Ratiometric contrast was defined as converted (B) divided by unconverted (A) components, which were derived by non-negative matrix factorization of fluorescence traces. At a power of 16 mW, the ratiometric contrast reached 472 in 200 s.

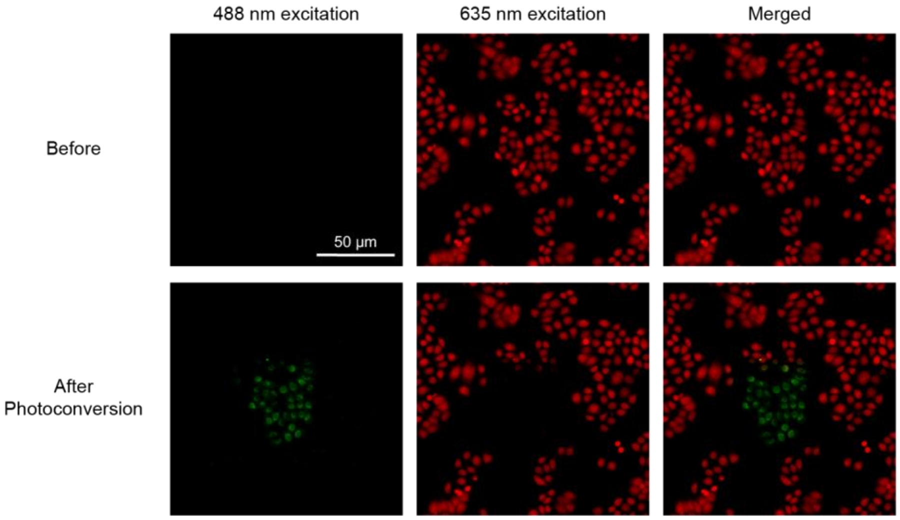

Figure S5 | Confocal fluorescence imaging of photoconverted cells. Confocal one-photon fluorescence images of SYTO62 stained HeLa cells before (top panels) and following (bottom panels) two-photon photoconversion. The two-color images were taken with an Olympus FV1000 confocal microscope using continuous-wave lasers at wavelengths of 488 nm (green) and 635 nm (red), respectively.

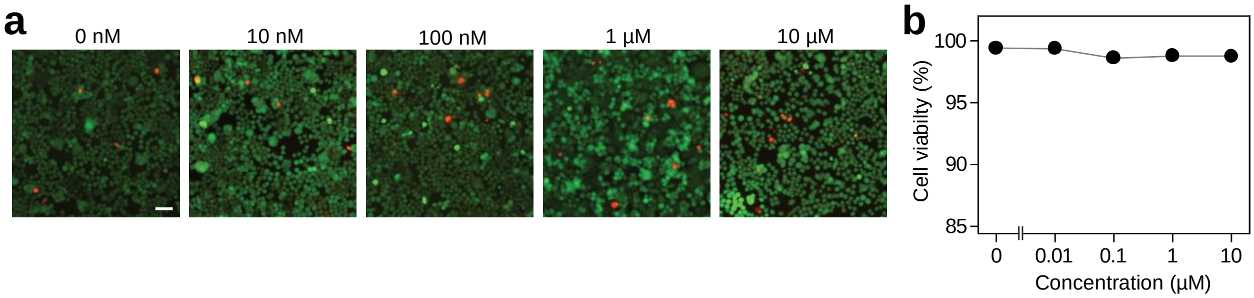

Figure S6| Cell viability after staining with SYTO62. a, Cell viability assay for HeLa cells 24 hours post staining with SYTO62 at various concentrations. Green indicates viable cells and red indicates dead cells. Scalebar, 50 µm. **b**, Quantification of cell viability.

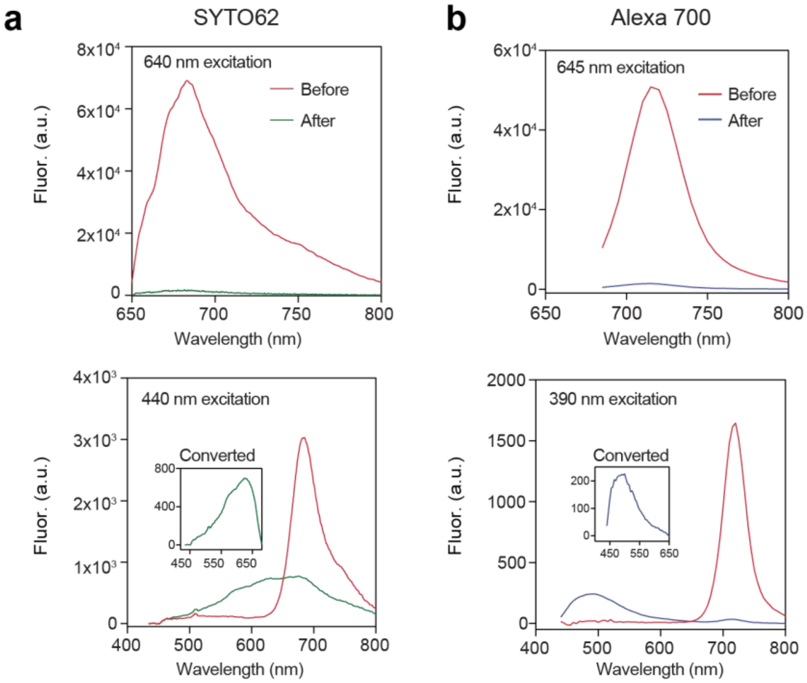

Figure S7 | One-photon photoconversion. SYTO62 and AlexaFluor 700 and after photoconversion with one-photon irradiation at 562-582 nm, 200 mW/mm^2 for up to 15 minutes. Here, a 45 µl sample of either 250 µM SYTO62 or 250 µM AlexaFluor 700 was irradiated with green light at high power until the sample was apparently bleached. **a**, Emission spectrum of SYTO62 at 640 nm excitation (top) and 440 nm excitation (bottom) are shown. Bottom inset shows approximate emission spectrum of converted dye following subtraction of unconverted dye emission. **b**, Emission spectrum of AlexaFluor 700 at 645 nm excitation (top) and 390 nm excitation (bottom) are shown. Bottom inset shows approximate emission spectrum of converted dye following subtraction of unconverted dye emission.

Supplementary Videos

Movie S1 | Real-time photoconversion of RAW 264.7 cells.

Movie S2 | 3D optical writing by two-photon excited photoconversion in SYTO62 mixed, transparent, UV-cured optical adhesive. The letters, ‘Y’, ‘U’, and ‘N’ are written on different planes separated by ~ 8.5 µm and z-stack images were taken with 1 µm spacing. Green channel fluorescence is reconstructed with ZEN (Zeiss).
